# Current Status of Hospitalist Practice and Factors Influencing Job Satisfaction in Korea

**DOI:** 10.1007/s11606-024-08910-8

**Published:** 2024-07-22

**Authors:** Song Yi Song, Hee Youn Han, Se Yoon Park, Jaewoong Kim, Kyung Mee Park, Taeyoung Kyong

**Affiliations:** 1 Department of Hospital Medicine, Yongin Severance Hospital, Yonsei University College of Medicine, Yongin, Korea; 2 Department of Internal Medicine, Hanyang University College of Medicine, Seoul, Korea; 3 Department of Biomedical Systems Informatics, Yonsei University College of Medicine, Seoul, Korea

**Keywords:** hospitalists, hospital medicine, mentors, job satisfaction, research

## Abstract

**Background:**

Although the roles and responsibilities of hospitalists have grown considerably in recent years, research on the current job status and satisfaction levels of Korean hospitalists is lacking.

**Objective:**

We investigate the present state of Korean hospitalists and the factors influencing their job satisfaction 6 years after the pilot program’s launch.

**Design:**

This cross-sectional analysis was based on an online survey conducted from January 30 to February 18, 2023.

**Participants:**

Korean hospitalists (*N* = 303)

**Main Measures:**

The survey encompassed participant demographics, hospital information, education, clinical practice, research involvement, and job satisfaction. We employed multiple logistic regression analyses to identify determinants of satisfaction as a hospitalist.

**Key Results:**

The analysis was based on 79 hospitalists’ responses (response rate 26%). Respondents had a median age of 39 years; approximately half were male internal medicine specialists, possessing over 3 years of hospitalist experience. Most respondents were interested in clinical work (94.4%), with only 21.5% interested in research and evidence-based medicine. Over two-thirds indicated that non-clinical duties occupied less than 20% of their time. Overall, job satisfaction among hospitalists averaged 51.9%. Notably, the availability of a research mentor was significantly associated with job satisfaction (*P* = .011). While hospitalists with more than 3 years of experience, more hospitalists per facility, and autonomy were associated with increased job satisfaction, these associations were not statistically significant. Furthermore, there was no association between night shift work, work type, or work hours and job satisfaction.

**Conclusions:**

Although Korean hospitalists primarily focus on clinical practice, our study underscores the positive impact of mentorship from research mentors on job satisfaction, supported by comprehensive univariate and multivariate analyses. These findings signal a progressive transformation in the role of Korean hospitalists, as they increasingly engage in research alongside patient care.

**Graphical Abstract:**

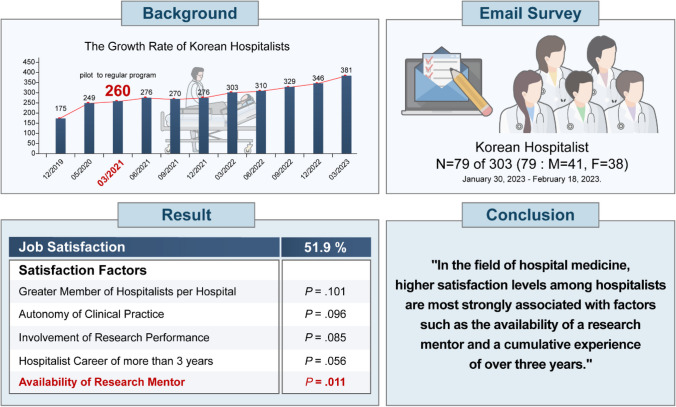

**Supplementary Information:**

The online version contains supplementary material available at 10.1007/s11606-024-08910-8.

## INTRODUCTION

The field of hospital medicine has experienced rapid growth as a medical specialty. Since the establishment of the hospitalist system in the United States in 1996, the number of hospitalists has significantly increased, reaching over 50,000 by 201.^1^ Numerous studies have demonstrated that the inpatient care provided by hospitalists has been effective in improving treatment outcomes, such as reducing the length of hospital stay,^2–7^ mortality rates,^8^ 30-day readmission rates,^9^ total hospital costs ^4,7,9^ and increased patient satisfaction,^5–7^ while also enhancing communication among healthcare professionals.^10^

In Korea, trainee residents traditionally manage inpatient treatment, resulting in a heavy workload for residents. In response to this challenge, legislation aimed at improving the training environment and status of residents was enacted in December 2015. In December 2017, this legislation reduced the maximum weekly training hours from approximately 110 to 88 h. However, the decrease in residents’ working hours raised concerns about patient safety. As public expectations for healthcare quality have risen, there has been a growing demand for a shift from a resident-centered approach to a specialist-centered practice in inpatient care.^11,12^

To strengthen the safety and efficiency of inpatient management, and to address the healthcare disparities by improving the training environment for the resident, a pilot project dedicated to the Korean hospitalist system was initiated in September 2016 to introduce the concept of “Inpatient Specialist” (or hospitalists) in Korea. Since being a hospitalist requires board certification as a basic qualification, specialists from various fields have been able to apply, allowing for a wide array of specialties to be included. The pilot project transitioned to a full-fledged program in January 2021, resulting in a total of 303 hospitalists in 162 wards across 56 medical centers as of March 2022.

To effectively establish the hospitalist system and promote informed career choices in hospital medicine, it is crucial to have up-to-date information about the current state of the hospitalist system in Korea. Additionally, to ensure the stable development of the hospitalist system, it is essential to comprehensively investigate both inhibiting and facilitating factors. To achieve these objectives, we conducted a survey to assess the current status of hospitalists in Korea and their job satisfaction 6 years after the initiation of the pilot project.

## METHODS

### Study Design and Population

This cross-sectional analysis was based on an online survey conducted from January 30 to February 18, 2023, over 20 days. The survey targeted all physicians with a hospitalist role in Korea (*N* = 303) who are registered members of the Korea Society of Hospital Medicine and the Korean Society of Surgical Hospital Medicine. A link to the online survey was sent to prospective participants via text messages and e-mails, with reminders being sent on the 4th, 10th, and 14th days to encourage participation. The respondents were asked to provide identification numbers to prevent duplicate responses, as their data were anonymized. Only one response per participant was accepted. This survey research was conducted following the Checklist for Reporting of Survey Studies (CROSS) guidelines.^13^

### Ethical Considerations

This study was approved by the Institutional Review Board of Yongin Severance Hospital and the study protocol adheres to the tenets of the Declaration of Helsinki (approval number 9-2022-0176, approved on January 11, 2023). Written informed consent was obtained online from all participants.

### Survey Items

The survey encompassed several sections, including basic participant information (sex, age, marital status, presence of children, graduation year, specialty training, clinical instructor training, hospitalist experience, and current position), hospital details (type of medical institution, size, location, number of hospitalists, type of work schedule [type 1, 2, and 3], and department affiliation), motivation for becoming a hospitalist, participation in non-clinical activities (education, research, quality improvement, committee activities, etc.), education (3 items), clinical practice (12 items), research (7 items), job satisfaction (9 items), after-tax income, intention to continue working as a hospitalist, reasons for continuing in or leaving the job, and suggestions for boosting and stabilizing the hospitalist system. The types of work schedules are as follows: Type 1 is 5 days a week (daytime only), Type 2 is 7 days a week (daytime only), and Type 3 is 7 days a week (day and nighttime). We developed the survey questionnaire by referring to several literature sources.^14–16^

The motivation for becoming a hospitalist, job satisfaction, after-tax income, and intention to continue working as a hospitalist was assessed using a 5-point Likert scale, where responses rated four or higher were considered positive. To simplify the analysis, certain variables underwent dichotomization from the Likert 5-point scale to a binary scale. Specifically, regarding job satisfaction, responses such as “Very satisfied,” “Satisfied,” “Neutral,” “Dissatisfied,” and “Very dissatisfied” were categorized. For analysis purposes, responses indicating “Very satisfied” and “Satisfied” were merged into the “Satisfied” category, while responses of “Neutral,” “Dissatisfied,” and “Very dissatisfied” were grouped as “Dissatisfied.” Participants were presented with multiple options and could select all that applied regarding reasons for continuing or leaving the job. To provide suggestions for boosting and stabilizing the hospitalist system, participants were presented with a list of options and were asked to rank them in order of importance. We provided the survey questionnaire used in this survey study in Appendix 6.

### Statistical Analysis

Statistical analysis was performed using SPSS 26 and R software (version 4.3.0; https://www.r-project.org/). Categorical variables were presented as numbers and percentages whereas continuous variables were listed as median and interquartile range (IQR). To identify variables for inclusion in the multiple logistic regression model, univariate analyses were initially conducted for each variable, and subsequent multiple logistic regression analysis was performed using variables with a *P*-value below 0.2. To evaluate non-response bias, the difference between early responders and late responders was tested for each variable (Appendixes 3–5). Normality tests were conducted before testing continuous variables; as most of them did not satisfy normality, the Mann–Whitney *U* test was used. For the categorical variables, *P*-values were obtained using the chi-square test, and when the cell with the expected frequency of less than 5 was less than 20% of the total cells, the *P*-values obtained through Fisher’s exact test were used. Significance was set at .05.

## RESULTS

### Respondents’ Characteristics

**Figure 1:**
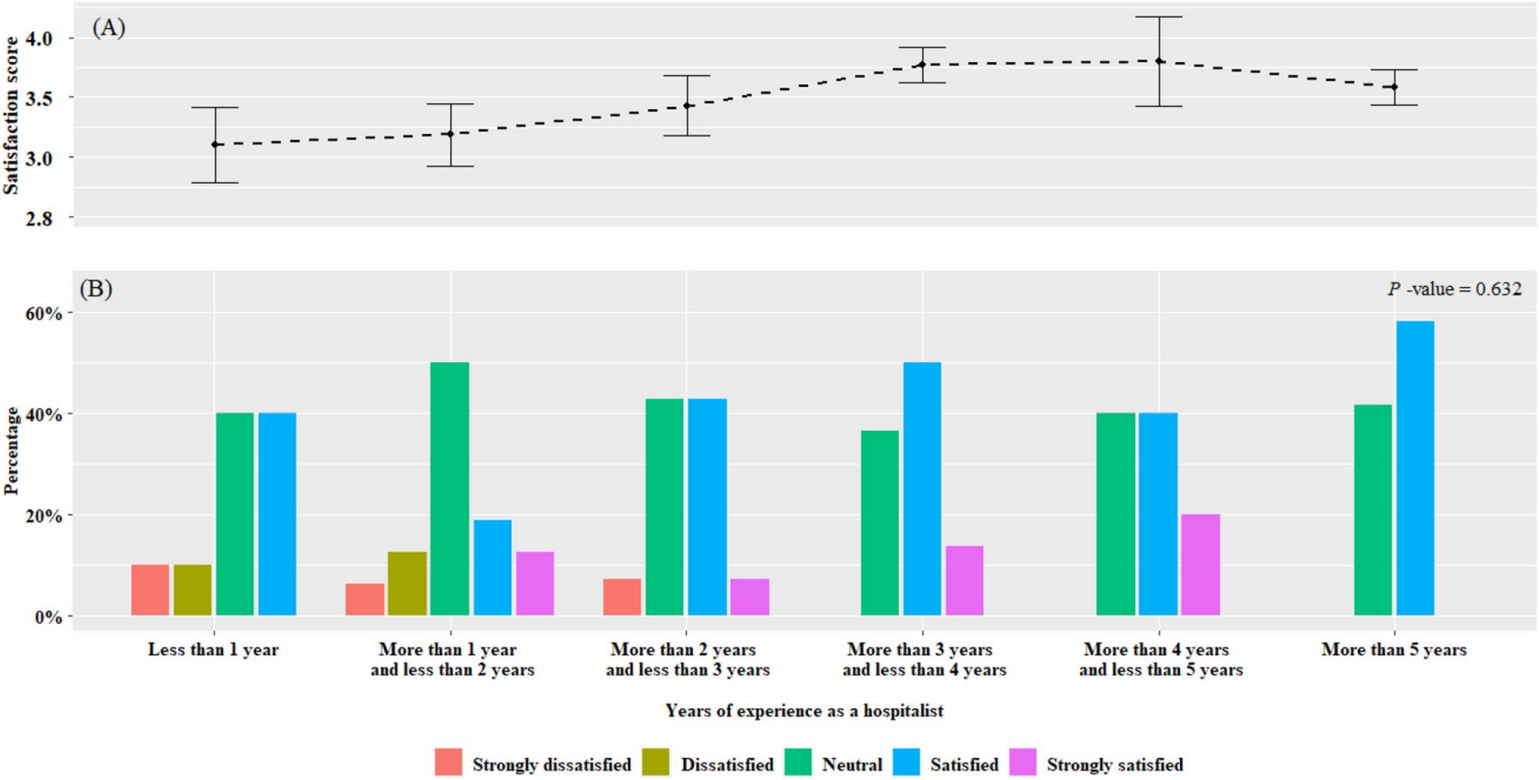
Job satisfaction based on years of experience as hospitalists. (**A**) Satisfaction levels rise with increasing experience, peaking at over 3 years. Hospitalists with less than 1 year of experience exhibit the lowest average satisfaction scores, while satisfaction slightly decreases for those with more than 5 years of experience. (**B**) The graph illustrates job satisfaction levels among respondents based on years of experience as hospitalists. The number of respondents reporting “satisfied” (blue) or “strongly satisfied” (purple) tends to increase with greater experience.

The median age of respondents was 39 years, with 51.9% being men. Of the total 79 respondents, 39 (49.4%) had over 3 years of experience as hospitalists (Fig. 1). Approximately 88.6% of respondents worked in the Seoul and Gyeonggi areas, and about two-thirds were employed in hospitals with more than 600 beds and tertiary hospitals (Table 1). A total of 28 respondents (35.4%) worked in independent hospitalist departments. Table 2 presents the characteristics of the hospitalists’ work scenario. The median number of inpatients was 15, which included an average of four new or transferred patients treated daily. The median weekly working hours were 45 h. One-third (35.4%) of the respondents worked night shifts, averaging 14 h per week. Most respondents worked in departmental or integrated type hospitalist services, with type 1 (51.9%) being the most common, followed by type 3 (25.3%) and type 2 (24.1%). Clinical nurse assistant participation was reported by 55.7%, while residents’ participation was reported by only 19%.

**Table 1:** Characteristics of Respondents in the Korean Hospitalist Survey

Characteristics	Respondents (*n* = 79)	Satisfied group (*n* = 40)	Dissatisfied group (*n* = 39)	*P*
Age, median (IQR)	39 (36–45)	41 (36–46)	39 (35–43)	.212
Male	41 (51.9)	23 (56.1)	18 (43.9)	.313
Married	58 (73.4)	30 (51.7)	28 (48.3)	.747
Have children	52 (65.8)	27 (51.9)	25 (48.1)	.750
Year of graduation, median (IQR)	2010 (2005–2014)	2009 (2003–2013)	2011 (2007–2013)	.248
Trainee department				.993
Internal medicine	43 (54.4)	21 (48.8)	22 (51.2)	
Surgery	14 (17.7)	7 (50.0)	7 (50.0)	
Pediatrics	8 (10.1)	5 (62.5)	3 (37.5)	
Family medicine	6 (7.6)	3 (50.0)	3 (50.0)	
Obstetrics and gynecology	2 (2.5)	1 (50.0)	1 (50.0)	
Others*	6 (7.6)	3 (50.0)	3 (50.0)	
Trainee experience as a clinical instructor	54 (68.4)	27 (50.0)	27 (50.0)	.869
Years of experience as a hospitalist				
Less than 3 years	40 (50.6)	16 (40.0)	24 (60.0)	.056
Over 3 years	39 (49.4)	24 (61.5)	15 (38.5)	
Position				.455
Professor	4 (5.1)	1 (25.0)	3 (75.0)	
Associate professor	10 (12.7)	7 (70.0)	3 (30.0)	
Assistant professor	18 (2.3)	10 (52.6)	9 (47.4)	
Others (clinical professor, etc.)	46 (5.8)	22 (47.8)	24 (52.2)	
Classification of hospitals				.941
Tertiary	55 (69.5)	28 (50.9)	27 (49.1)	
General	24 (30.4)	12 (50.0)	12 (50.0)	
Size of hospitals				.814
1200 beds or more	28 (35.4)	15 (53.6)	13 (46.4)	
900–1200 beds	13 (16.5)	5 (38.5)	8 (61.5)	
600–900 beds	30 (38.0)	16 (53.3)	14 (46.4)	
Less than 600 beds	8 (10.1)	4 (50.0)	4 (50.0)	
Location				.925
Seoul metropolitan city	39 (49.4)	20 (51.3)	19 (48.7)	
Gyeonggi Province	31 (39.2)	16 (51.6)	15 (48.4)	
Others	9 (11.4)	4 (44.4)	5 (55.6)	
Number of hospitalists per hospital, median (IQR)	16 (5–23)	22 (8–23)	9 (3–23)	.053
Classification of affiliation				.467
General internal medicine or surgery	35 (44.3)	15 (42.9)	20 (57.1)	
Department of hospital medicine	28 (35.4)	16 (57.1)	12 (42.9)	
Individual department	16 (20.3)	9 (56.3)	7 (43.8)	
Annual income				.401
50–100 million won	6 (7.6)	2 (33.3)	4 (66.7)	
100–150 million won	54 (68.4)	26 (48.1)	28 (51.9)	
150–200 million won	19 (24.1)	12 (63.2)	7 (36.8)	

Data are expressed as numbers (%) unless otherwise specified

*IQR* interquartile range. *One representative for each from the following: anesthesiology and pain medicine, radiology and oncology, neurology, neurosurgery, emergency medicine, and psychiatry and behavioral sciences

**Table 2:** Characteristics of Hospitalist Work in Korea

Characteristics	Respondents (*n* = 79)	Satisfied group (*n* = 40)	Dissatisfied group (*n* = 39)	*P*
Type of hospital ward				NA
Departmental	39 (49.4)	22 (56.4)	17 (43.6)	
Integrated	38 (49.1)	18 (47.4)	20 (52.6)	
Acute	11 (13.9)	3 (27.3)	8 (72.7)	
Type of work schedule				.900
Type 1 (daytime only, 5 days a week)	41 (51.9)	20 (48.8)	21 (51.2)	
Type 2 (daytime only, 7 days a week)	18 (22.8)	9 (50.0)	9 (50.0)	
Type 3 (day and nighttime, 7 days a week)	20 (25.3)	11 (55.0)	9 (45.0)	
Mean daily number of new admissions, median (IQR)	4 (3–5)	4 (3–5)	5 (3–6.25)	.493
Mean daily number of inpatients, median (IQR)	15 (12–20)	15 (12.75–18.5)	15 (12.75–20)	.949
Maximum number of inpatients, median (IQR)	19 (15–23)	19 (15–23)	18.5 (15–23)	.421
Average working hours in a week, median (IQR)	45 (40–50)	42 (40–45)	45 (40–50)	.300
Night duty status	28 (35.4)	14 (50.0)	14 (50.0)	.934
Night work hours in a week, median (IQR)	14 (9–19.2)	0 (0–12.5)	0 (0–11.05)	.708
Involvement of physician assistant nurses	44 (55.7)	25 (56.8)	19 (43.2)	.266
Involvement of residents	15 (19)	6 (40.0)	9 (60.0)	.360
Autonomy				.096
High	48 (60.8)	29 (60.4)	19 (39.6)	
Moderate	20 (25.3)	7 (35.0)	13 (65.0)	
Low	11 (13.9)	4 (36.4)	7 (63.6)	
Proportion of non-clinical tasks in addition to patient care				
20% or less	59 (74.7)	29 (49.2)	30 (50.8)	.315
20–40%	17 (21.5)	8 (47.1)	9 (52.9)	
40–60%	3 (3.8)	3 (100.0)	0	
Participation in education	59 (74.7)	28 (47.4)	31 (52.5)	.439
Participation in research	34 (43.0)	21 (61.8)	13 (38.2)	.085
Availability of research mentor	14 (17.7)	12 (85.7)	2 (14.3)	.004

Data are expressed as numbers (%) unless otherwise specified

*IQR* interquartile range, *NA* not available

### Non-clinical Work (Education, Research)

Over two-thirds of the respondents indicated that they participated in non-clinical work for less than 20% of their time. Among them, 59 individuals (74.7%) reported being involved in education, with the majority focusing on teaching nurses, administrators, and physicians. Clinical topics were the most common (98.3%), followed by procedures, communication, and point-of-care ultrasound. Over the past 3 years, 34 (43.0%) respondents participated in research, while 14 (17.7%) served as project leaders. Additionally, 24 (30.4%) respondents reported publishing papers, while only 16.5% had a research mentor (Table 2).

### Motivations and Job Satisfaction

The most prevalent motivation for becoming a hospitalist was “new opportunity as a hospitalist” (30.4%), followed by “financial stability” (21.5%). Most interests focused on clinical work, with quality improvement and education also garnering attention at 39.2% and 30.2%, respectively. Interest in research and evidence-based medicine was at 21.5% (Supplemental Table 1). A total of 32 (40.5%) respondents were involved in hospital committees, while 22.8% were involved in quality improvement activities. Overall job satisfaction as a hospitalist was at 51.9%, with 54.4% expecting future satisfaction to increase. Satisfaction with personal and family leisure time was 74.9%, but satisfaction with support from hospital management and hospitalist leaders was 49.4% and 29.1%, respectively. While satisfaction with relationships with nurses and hospitalists was 75.9% and 68.4%, respectively, satisfaction with other professionals was 49.4% (Table 3).

**Table 3:** Survey Results of Hospitalists’ Job Satisfaction and Related Factors

Characteristics	Respondents (*n* = 79)
Satisfaction with non-clinical work	31 (39.2)
Match with expected job responsibilities	43 (54.4)
Satisfaction as a hospitalist	41 (51.9)
Satisfaction regarding work–life balance	
Personal/family time	59 (74.7)
Workload outside of work	39 (49.4)
Satisfaction regarding support from hospitalist leader	
Support from management	39 (49.4)
Support from hospitalist leader	23 (29.1)
Satisfaction regarding relationship	
Nurses	60 (75.9)
Hospitalists	54 (68.4)
Other professionals	39 (49.4)
Expectations for increased job satisfaction	43 (54.4)

Data are expressed as numbers (%)

### Factors Influencing Satisfaction as a Hospitalist

Of the 40 respondents who reported being satisfied or strongly satisfied as hospitalists, several variables exhibited *P*-values less than .20, including experience as a hospitalist of more than 3 years (*P* = .056), the number of hospitalists per hospital (*P* = .101), autonomy (*P* = .096), presence of research performance (*P* = .085), availability of a research mentor (*P* = .004), and curiosity and interest in hospital medicine (*P* = .144). Figure 1 presents the responses related to job satisfaction based on years of experience as a hospitalist. The multiple logistic regression analysis revealed that the availability of a research mentor significantly influenced satisfaction as a hospitalist (*P* = .011) (Table 4).

**Table 4:** Multiple Logistic Regression Analysis for Satisfaction as a Hospitalist

Characteristics	*B*	S.E.	Odds ratio	95% CI	*P*
Years of experience as a hospitalist					
Less than 3 years	Ref.				
Over 3 years	0.718	0.570	2.050	0.671–6.266	.208
Number of hospitalists per hospital	0.029	0.024	1.03	0.983–1.079	.214
Autonomy					.514
High	−0.297	0.859	0.743	0.138–4.002	.730
Moderate	−0.955	0.954	0.385	0.059–2.497	.317
Low	Ref.				
Research performance					
Yes	−0.108	0.634	0.898	0.259–3.11	.865
No					
Availability of research mentor					
Yes	2.624	1.032	13.795	1.824–104.34	.011
No	Ref.				
Curiosity and interest in hospital medicine					
Yes	0.925	0.561	2.522	0.84–7.572	.099
No	Ref.				
Annual income					.319
50–100 million won	Ref				
100–150 million won	1.287	1.26	3.620	0.306–42.780	.307
Over or equal to 150 million won	1.928	1.341	6.875	0.496–95.283	.151

*CI* confidential interval

### Continued Employment

Most respondents were either undecided or planning to continue working as hospitalists, with only seven individuals indicating that they had no intention to continue. The top factors for continuing employment were ranked as follows: work–life balance, job stability, and satisfaction with professional expertise. The primary reason for not continuing was salary, followed by social status and recognition, and job stability (Supplemental Table 2).

## DISCUSSION

The effectiveness of the hospitalist system has been well-documented both in Korea and internationally, with notable improvements in patient and staff satisfaction, reduced length of stay, lower total hospital costs, decreased readmission rates, reduced rates of unplanned emergency department visits within 30 days of discharge, and fewer hospital-related adverse events.^3,5–10,17,18^ Although 7 years have passed since the inception of the hospitalist system in Korea, its growth has remained relatively slow compared to similar systems in the United States;^1,19^ this is despite the numerous positive effects demonstrated following its introduction.

This study analyzed job satisfaction and its implications for the establishment of the hospitalist system comprising inpatient specialists. The overall job satisfaction rate among participants was 51.9%. Notably, the presence of research mentorship significantly influenced job satisfaction, a finding consistent with prior research demonstrating the positive impact of effective mentorship on hospitalist job satisfaction.^20,21^ However, this study specifically focused on research mentorship and did not explore the presence of mentors in other aspects of hospitalist practice; therefore, questions regarding the significance of mentorship are outside the current research scope.

Despite the prevalence of research mentorship, most respondents (94.4%) expressed a primary interest in clinical work, with only 21.5% showing interest in research and evidence-based medicine. This suggests that Korean hospitalists predominantly concentrate on clinical responsibilities. Given the observed impact of research mentorship on job satisfaction, it is reasonable to anticipate that enhancing opportunities for hospitalists to integrate both clinical and research roles, along with access to research mentors, could boost overall job satisfaction. However, further investigation is warranted to comprehensively assess mentorship status and satisfaction across various aspects of hospitalist practice.

Job satisfaction exhibited a positive correlation with years of experience as an inpatient hospitalist; however, this association did not demonstrate statistical significance owing to the limited sample size. These findings align with those of an analysis of healthcare claims data in the United States, which indicated that first-year hospitalists had higher in-hospital mortality rates compared to those with more experience.^22^ This suggests that higher experience could mitigate patient-related stress, potentially leading to greater satisfaction. Moreover, reduced patient care burdens could contribute to enhanced satisfaction by affording hospitalists more leisure time for personal pursuits and family activities. However, future studies must consider potential biases, such as the possibility that less satisfied individuals could change jobs earlier in their careers, while more satisfied hospitalists could continue practicing in the institution for a longer duration.

Our study also found that the number of hospitalists per hospital can influence job satisfaction. A higher concentration of hospitalists within a single institution offers advantages such as increased work schedule flexibility and reduced burdens in areas like critical care.^23^ Furthermore, having a greater number of hospitalists facilitates collaborative quality improvement initiatives and provides opportunities for training physicians, physician assistants, and nurses.^24^ This collaborative approach not only alleviates workload but also improves overall quality of life and expands avenues for research and training. Additionally, when multiple hospitalists work within the same hospital, they can serve as role models for each other.

In Korea, hospitalists typically work under three shift types, each with varying preferences based on clinical experience and individual inclinations. Consequently, no single shift type is universally superior to another. If an adequate number of hospitalists is available, hospitals can select and operate shifts based on considerations such as faculty satisfaction, hospital profitability, and the satisfaction of patients and colleagues.

Contrary to our expectations, no significant associations were found between job satisfaction and factors such as night shift work, work type, or work hours. While excessive hospitalization work could theoretically impact job satisfaction,^25^ it is important to note that the Korean hospitalist system is relatively new, with over half the respondents having less than 3 years of experience as hospitalists. Therefore, further research is needed to explore the effects of work type and intensity, including night shifts, on hospitalist job retention and satisfaction.

In contrast to previous studies linking financial compensation with hospitalist job satisfaction, this study did not reveal any significant differences in job satisfaction based on annual income. This suggests that other factors beyond financial compensation play a pivotal role. Recent research has identified the likelihood of faculty appointment as an independent predictor of hospitalist career continuation,^26^ underscoring the multifaceted nature of the job satisfaction determinants.

The study limitations include a relatively low response rate of 26%, which implies that the findings may not fully represent the current perspectives and opinions of hospitalists in Korea. Secondly, we conducted the analysis after converting the 5-point Likert scale for satisfaction into a binary variable, which may lead to a possible information loss and reduced statistical power. Lastly, as the hospitalist system matures, the factors influencing job satisfaction could evolve. Therefore, conducting regular surveys at approximately 3-year intervals with a more substantial participant pool in the future could provide more comprehensive insights and contribute to system stability.

Nonetheless, this study is significant as it is a timely examination of job satisfaction among hospitalists during the early stages of the development of Korea’s hospitalist system.

## CONCLUSION

To further enhance the success of Korea’s hospitalist system, it is imperative to prioritize the establishment of mentorship programs, as identified in this study, and increase the employment of hospitalists within healthcare institutions. Achieving these objectives requires setting realistic pricing structures for hospitalists to enable cost-effective hiring by institutions, along with recognizing hospitalists as faculty members and providing them with research opportunities.

## Supplementary Information

Below is the link to the electronic supplementary material.

Supplementary file1 (PDF 352 KB)

## Data Availability

The dataset supporting the conclusions of this study is included as supplemental tables (raw data used in the analysis).
