# Supplementary material for: Current Status of Hospitalist Practice and Factors Influencing Job Satisfaction in Korea
Source: J Gen Intern Med. 2024 Jul 22;40(2):302–8. doi: 10.1007/s11606-024-08910-8 (PMC11803046; doi:10.1007/s11606-024-08910-8)
Supplement: Supplementary file 1 — Supplementary file1 (PDF 352 KB) [file 11606_2024_8910_MOESM1_ESM.pdf]

Appendix 1

Supplemental Table 1. Motivation for applying as a hospitalist and areas of interest

Characteristics	Respondents (n = 79)
Motivation for applying	
New opportunity as a hospitalist	24 (30.4)
Financial stability	17 (21.5)
Lack of interest in other fields	12 (15.2)
Curiosity and interest in hospital medicine	11 (13.9)
Areas of interest	
Clinical work	73 (92.4)
Quality improvement	31 (39.2)
Education	24 (30.4)
Research and evidence-based medicine	17 (21.5)

Data are expressed as numbers (%).

Appendix 2

Supplemental Table 2. Survey results on the intentions and reasons for hospitalists to continue or discontinue their employment.

Characteristics	Respondents (n = 79)
Job continuation	
Yes	45 (57.0)
Unsure	27 (34.2)
No	7 (8.9)
Reasons for job continuation	
Work-life balance	55 (69.6)
Job stability	29 (36.7)
Satisfaction with professional expertise	23 (29.1)
Reasons for not continuing	
Salary	23 (29.1)
Social status and recognition	17 (21.5)
Job stability	16 (20.3)
Frequent night duty	15 (19.0)
Lack of sense of accomplishment	15 (19.0)
Working environment	13 (16.5)
Relationship with medical staff	12 (15.2)
Staff shortage	11 (13.9)

Data are expressed as numbers (%).

Appendix 3

Supplemental Table 3. Characteristics of respondents in the Korean hospitalist survey

Characteristics	Respondents (n = 79)	Early responders (n=56)	Late responders (n=23)	*P*
Age, median (IQR)	39 (36–45)	39 (36-44)	41 (38-47)	0.198
Male	41 (51.9)	32 (57.1)	9 (39.1)	0.145
Married	58 (73.4)	42 (75.0)	16 (69.6)	0.619
Have children	52 (65.8)	37 (66.1)	15 (65.2)	0.942
Year of graduation, median (IQR)	2010 (2005–2014)	2011 (2006-2014)	2009 (2002-2013)	0.153
Trainee department				0.300
Internal medicine	43 (54.4)	32 (57.1)	11 (47.8)	
Surgery	14 (17.7)	11 (19.6)	3 (13.0)	
Pediatrics	8 (10.1)	5 (8.9)	1 (13.0)	
Family medicine	6 (7.6)	5 (8.9)	1 (4.3)	
Obstetrics and gynecology	2 (2.5)	1 (1.8)	1 (4.3)	
Others*	6 (7.6)	2 (3.6)	4 (17.4)	
Trainee experience as a clinical instructor	54 (68.4)	35 (62.5)	19 (82.6)	0.081
Years of experience as a hospitalist				0.071
Less than 3 years	40 (50.6)	32 (57.1)	8 (34.8)	
Over 3 years	39 (49.4)	24 (42.9)	15 (65.2)	
Position				0.821
Professor	4 (5.1)	3 (5.4)	1 (4.3)	
Associate professor	10 (12.7)	6 (10.7)	4 (17.4)	
Assistant professor	18 (2.3)	13 (23.2)	6 (26.1)	
Others (clinical professor, etc.)	46 (5.8)	34 (60.7)	12 (52.2)	
Classification of hospitals				0.586
Tertiary	55 (69.5)	40 (71.4)	15 (65.2)	
General	24 (30.4)	16 (28.6)	8 (34.8)	
Size of hospitals				0.185
1200 beds or more	28 (35.4)	19 (33.9)	9 (39.1)	
900–1200 beds	13 (16.5)	8 (14.3)	5 (21.7)	
600–900 beds	30 (38.0)	25 (44.6)	5 (21.7)	
Less than 600 beds	8 (10.1)	4 (7.1)	4 (17.4)	
Location				0.220
Seoul metropolitan city	39 (49.4)	29 (51.8)	10 (43.5)	
Gyeonggi Province	31 (39.2)	19 (33.9)	12 (52.2)	
Others	9 (11.4)	8 (14.3)	1 (4.3)	

Number of hospitalists per hospital, median (IQR)	16 (5–23)	17 (4-26)	15 (9-22)	0.553
Classification of affiliation				0.837
General internal medicine or surgery	35 (44.3)	26 (46.4)	9 (39.1)	
Department of hospital medicine	28 (35.4)	19 (33.9)	9 (39.1)	
Individual department	16 (20.3)	11 (19.6)	5 (21.7)	
Annual income				0.469
50–100 million won	6 (7.6)	5 (8.9)	1 (4.3)	
100–150 million won	54 (68.4)	36 (64.3)	18 (78.3)	
150–200 million won	19 (24.1)	15 (26.8)	4 (17.4)	

Data are expressed as numbers (%) unless otherwise specified.

IQR: interquartile range.

Appendix 4

Supplemental Table 4. Characteristics of hospitalist work in Korea

Characteristics	Respondents (n = 79)	Early responders (n=56)	Late responders (n=23)	*P*
Type of hospital ward				NA
Departmental	39 (49.4)			
Integrated	38 (49.1)			
Acute	11 (13.9)			
Type of work schedule				0.797
Type 1 (daytime only, 5 days a week)	41 (51.9)	30 (53.6)	11 (47.8)	
Type 2 (daytime only, 7 days a week)	18 (22.8)	13 (23.2)	5 (21.7)	
Type 3 (day and nighttime, 7 days a week)	20 (25.3)	13 (23.2)	7 (30.4)	
Mean daily number of new admissions, median (IQR)	4 (3–5)	4 (3-6)	5 (3-5)	0.960
Mean daily number of inpatients, median (IQR)	15 (12–20)	15 (12.25-20)	15 (14-18)	0.987
Maximum number of inpatients, median (IQR)	19 (15–23)	20 (15-23)	18 (15-25)	0.926
Average working hours in a week, median (IQR)	45 (40–50)	42 (40-50)	45 (40-49)	0.542
Night duty status	28 (35.4)	20 (35.7)	8 (34.8)	0.937
Night work hours in a week, median (IQR)	14 (9–19.2)	14 (10.3-16.2)	12.5 (7.3-31)	0.784
Involvement of physician assistant nurses	44 (55.7)	29 (51.8)	15 (68.2)	0.189
Involvement of residents	15 (19)	11 (19.6)	4 (17.4)	>0.999
Autonomy				0.687
High	48 (60.8)	33 (58.9)	15 (65.2)	
Moderate	20 (25.3)	14 (25.0)	6 (26.1)	
Low	11 (13.9)	9 (16.1)	2 (8.7)	
Proportion of non-clinical tasks in addition to patient care				0.388
20% or less	59 (74.7)	43 (76.8)	16 (69.6)	
20–40%	17 (21.5)	10 (17.9)	7 (30.4)	
40–60%	3 (3.8)	3 (5.4)	0 (0.0)	
Participation in education	59 (74.7)	41 (73.2)	18 (78.3)	0.639
Participation in research	34 (43.0)	25 (44.6)	9 (39.1)	0.653
Availability of research mentor	14 (17.7)	9 (16.1)	5 (21.7)	0.535

Data are expressed as numbers (%) unless otherwise specified.

IQR: interquartile range; NA: not available

Appendix 5

Supplemental Table 5. Survey results of hospitalists' job satisfaction and related factors

Characteristics	Respondents (n = 79)	Early responders (n=56)	Late responders (n=23)	*P*
Satisfaction with non-clinical work	31 (50.8)	23 (53.5)	8 (44.4)	0.519
Match with expected job responsibilities	43 (54.4)	32 (57.1)	11 (47.8)	0.450
Satisfaction as a hospitalist	40 (50.6)	28 (50.0)	12 (52.2)	0.861
Satisfaction regarding work–life balance				
Personal/family time	59 (74.7)	44 (78.6)	15 (65.2)	0.215
Workload outside of work	39 (49.4)	28 (50.0)	11 (47.8)	0.861
Satisfaction regarding support from hospitalist leader				
Support from management	23 (29.1)	15 (26.8)	8 (34.8)	0.477
Support from hospitalist leader	39 (49.4)	24 (42.9)	15 (65.2)	0.071
Satisfaction regarding relationship				
Nurses	60 (75.9)	45 (80.4)	15 (65.2)	0.153
Hospitalists	54 (68.4)	39 (69.6)	15 (65.2)	0.701
Other professionals	39 (49.4)	32 (57.1)	7 (30.4)	0.031
Expectations for increased job satisfaction	38 (48.1)	27 (48.2)	11 (47.8)	0.975

Data are expressed as numbers (%).

Appendix 6

Survey Questionnaire

0. Consent to participate in the survey

Do you agree to participate in this survey (a survey of the current status of hospitalists and factors influencing job satisfaction in Korea)?

□ Yes □ No

1. Basic information for survey participants

1-1. Sex: □ Female □ Male

1-2. Age: (   ) years old

1-3. Married: □ Yes □ No

1-4. Do you have children? □ Yes □ No

1-5. What is your year of graduation from medical school? (   ) year

1-6. Please select your trainee department from the following options.

□ Internal medicine □ Surgery □ Family medicine □ Anesthesiology and Pain Medicine □ Radiation Oncology □ Pathology □ Urology □ Obstetrics and Gynecology □ Plastic Surgery □ Pediatrics □ Neurology □ Neurosurgery □ Ophthalmology □ Radiology □ Emergency Medicine □ Otorhinolaryngology □ Rehabilitation Medicine □ Psychiatry and Behavioral Sciences □ Orthopedic Surgery □ Laboratory Medicine □ Dermatology □ Nuclear Medicine □ Cardiothoracic Surgery

1-7. Do you have trainee experience as a clinical instructor (fellowship)?

□ Yes □ No

1-8. What type of fellowship training have you received?

- Internal Medicine □ Infectious Disease □ Endocrinology □ Rheumatology □ Gastroenterology □ Nephrology □ Cardiology □ Hematology-Oncology □ Pulmonology □ Allergy

- Pediatrics □ Pediatric Infectious Disease □ Pediatric Endocrinology □ Pediatric Rheumatology □ Pediatric Gastroenterology □ Pediatric Nephrology □ Pediatric Cardiology □ Pediatric Hematology-Oncology □ Pediatric Pulmonology and Allergy □ Neonatology □ Pediatric Neurology

- Surgery □ Upper Gastrointestinal Surgery □ Colorectal Surgery □ Hepatobiliary Surgery □ Thyroid Surgery □ Breast Surgery

1-9. How many years of experience do you have as a hospitalist?

□ Less than 1 year □ More than 1 year and less than 2 years □ More than 2 years and less than 3 years □ More than 3 years and less than 4 years □ More than 4 years and less than 5 years □ More than 5 years

1-10. What is your current position?

□ Professor □ Associate professor □ Assistant professor □ Clinical professor □ Clinical associate professor □ Clinical assistant professor □ Medical professor □ Other

2. Basic information about the hospital you work in

2-1. What type of hospital do you work at?

□ Tertiary general hospital □ General hospital □ Hospital □ Clinic

2-2. Which of the following hospitals do you work in?

□ Public University Hospital □ Private University Hospital □ Public Non-University Hospital □ Private Non-University Hospital

2-3. Please select the size of the hospital where you work.

□ Less than 300 beds □ 300–600 beds □ 600–900 beds

□ 900–1200 beds □ 1200 beds or more

2-4. Where is your work area?

□ Seoul □ Gyeonggi Province (including Incheon)

□ Gyeongsang Province (including Busan, Daegu, and Ulsan)

□ Jeolla Province (including Gwangju) □ Chungcheong Province (including Daejeon and Sejong)

□ Gangwon Province □ Jeju Island

2-5. What is the number of hospitalists in your hospital? (   )

2-6. What is your affiliation within the hospital where you work?

□ Department of Hospital Medicine □ Internal Medicine (General or Comprehensive)

□ Surgery (General or Comprehensive)

□ Specific Specialties (Hematology-Oncology, Neurosurgery, Obstetrics and Gynecology, etc.)

□ Other (   )

3. Motivation for applying as a hospitalist

3-1. Financial stability

□ Strongly agree □ Somewhat agree □ Neutral □ Somewhat disagree □ Strongly disagree

3-2. Curiosity and interest in hospital medicine

□ Strongly agree □ Somewhat agree □ Neutral □ Somewhat disagree □ Strongly disagree

3-3. Lack of interest in other fields

□ Strongly agree □ Somewhat agree □ Neutral □ Somewhat disagree □ Strongly disagree

3-4. New opportunity as a hospitalist

□ Strongly agree □ Somewhat agree □ Neutral □ Somewhat disagree □ Strongly disagree

4. Areas of interest and work areas

4-1. What are your primary areas of interest within hospital medicine? (Multiple responses)

□ Clinical work □ Education □ Research and evidence-based medicine □ Quality improvement □ None □ Other (   )

4-2. Do you engage in duties outside of clinical practice as a hospitalist? □ Yes □ No

4-3. If you participate in duties outside clinical practice, please select all that apply from the following options:

□ Quality improvement activities □ Hospital committees □ Research □ Education □ Other (   )

4-4. If you participate in duties outside clinical practice, how satisfied are you with these non-clinical responsibilities?

□ Very Satisfied □ Satisfied □ Neutral □ Dissatisfied □ Very Dissatisfied

4-5. Do you believe that your career as a hospitalist aligns with what you expected in terms of clinical practice and non-clinical duties before choosing this path?

□ Very much so □ Yes □ Neutral □ No □ Not at all

4-6. If you answered “No” or “Not at all” to Question 4-5, please provide the reasons for your response.

5. Education

5-1. Do you participate in education for staff, residents, or students?

□ Yes □ No

5-2. What is the target audience for your education? (Multiple responses)

□ Hospital staff (physicians, nurses, administrative staff, etc.) □ Residents/interns □ Students

5-3. What is the content of your education? (Multiple responses)

□ Clinical practice (diagnosis, treatment) □ Procedures □ Communication with medical team □ Point-of-care ultrasound □ Other ( )

6. Clinical practice

6-1. On average, how many new inpatient admissions (including transfer patients) have you handled in a day over the past month? ( )

6-2. What is the average number of inpatients you've treated within the past month? ( )

6-3. What is the maximum number of inpatients you've treated within the past month? ( )

6-4. In the past month, what percentage of your inpatient admissions were classified into specialized care disease groups, general care, or simple care categories?

□ Less than 20% □ 20–40% □ 40–60% □ 60–80% □ 80–100%

6-5. How many hours do you work in a week (Monday–Sunday)? [For example, if you work 10 hours a day for 5 days, it would be 50 hours] ( ) hours

6-6. Do you participate in night shifts? □ Yes □ No

6-7. What is the average number of hours you work during the night in a week (between 10 PM and 6 AM)? [For example, if you work approximately 2 hours a day for 3 days, it would be 6 hours] ( ) hours

6-8. What percentage of your duties involve research, staff management, and administrative activities?

□ Less than 20% □ 20–40% □ 40–60% □ 60–80% □ 80–100%

6-9. Does your hospital have physician assistants involved in the care of inpatients managed by hospitalists? □ Yes □ No

6-10. Does your hospital have residents involved in the care of inpatients managed by hospitalists? □ Yes □ No

6-11. What is your work schedule like?

□ 5 days a week (daytime only) □ 7 days a week (daytime only) □ 7 days a week (day and nighttime)

6-12. What is the type of hospital ward where you work?

- Departmental: Limits inpatient care to specific department patient groups.<br>- Integrated: Manages complex, critically ill patients collectively.<br>- Acute: Focuses on resolving issues within 72 hours for patients requiring emergency care.<br><br>* Reference: Kim HW. The current status of hospital medicine in Korea, 2019. Korean J Med. 2019;94(2):139–44.

□ Departmental □ Integrated □ Acute

6-13. What is the level of your job authority in clinical care?

□ Very high □ High □ Neutral □ Restricted □ Very restricted

7. Research

** Please respond as per research conducted in the last three years (2019–2022).

7-1. Have you been involved in research (academic presentations, paper writing, participation in multi-institutional studies, etc.)?

□ Yes □ No

7-2. Have you received research funding as a principal investigator to conduct research projects in the last three years (including ongoing projects)?

□ Yes □ No

7-3. If you have received research funding and are currently conducting research, which of the following categories best describes your research project? (Multiple responses)

□ Government-funded projects □ Industry-funded projects □ Institutional projects □ Other ( )

7-4. Have you had any experience as the first author (primary author or corresponding author) publishing in academic journals in the last three years? (Multiple responses)

□ None □ Published in SCI/SCIE journals □ Published in SCOPUS journals □ Published in KCI (Korean Citation Index) journals □ Published in other journals

7-5. How many journal articles do you read every week?

□ 0 articles □ 1–5 articles □ 6–10 articles □ 11–15 articles □ 16 or more articles

7-6. Have you attended domestic academic conferences since working as a hospitalist?

□ Yes □ No

7-7. Have you attended international academic conferences since working as a hospitalist?

□ Yes □ No

7-8. Do you have a research mentor?

□ Yes □ No

8. Satisfaction

8-1. Are you satisfied as a hospitalist?

□ Very satisfied □ Satisfied □ Neutral □ Dissatisfied □ Very dissatisfied

8-2. Do you think your job satisfaction will improve in the next 5 years compared to now?

□ Very much so □ Yes □ Neutral □ Not really □ Not at all

8-3. Are you satisfied with the time you spend by yourself and with your family while working as a hospitalist?

□ Very satisfied □ Satisfied □ Neutral □ Dissatisfied □ Very dissatisfied

8-4. Are you satisfied with the workload outside regular working hours?

□ Very satisfied □ Satisfied □ Neutral □ Dissatisfied □ Very dissatisfied

8-5. Are you satisfied with the support from the hospital management?

□ Very satisfied □ Satisfied □ Neutral □ Dissatisfied □ Very dissatisfied

8-6. Are you satisfied with the support from the hospitalist leader?

□ Very satisfied □ Satisfied □ Neutral □ Dissatisfied □ Very dissatisfied

8-7. Are you satisfied with your relationships with fellow hospitalists?

□ Very satisfied □ Satisfied □ Neutral □ Dissatisfied □ Very dissatisfied

8-8. Are you satisfied with your relationships with non-hospitalist physicians?

□ Very satisfied □ Satisfied □ Neutral □ Dissatisfied □ Very dissatisfied

8-9. Are you satisfied with your relationships with nurses?

□ Very satisfied □ Satisfied □ Neutral □ Dissatisfied □ Very dissatisfied

9. Other

9-1. What is your annual income (after taxes)?

□ Less than 50 million won □ 50–100 million won □ 100–150 million won □ 150–200 million won □ Over 200 million won

9-2. Do you intend to continue working as a hospitalist in the future?

□ Yes □ No □ Not sure

9-3. If you plan to continue working as a hospitalist, what are your reasons? (Select all that apply)

□ Job stability □ Satisfaction with professional expertise □ Work-life balance

□ Other ( )

9-4. If you do not intend to continue working as a hospitalist, what are your reasons? (Select all that apply)

□ Excessive workload □ Salary □ Staff shortage □ Frequent night duty □ Relationship with medical staff □ Job stability □ Social status and recognition □ Lack of sense of accomplishment □ Working environment

□ Other ( )

10. Please select the most important ones in order of priority for the activation and stability of the hospitalist system.

□ Improvement of low level of reimbursement to providers

□ Flexibility in hospitalist work regulations (improvement of rigid regulations such as restricted practice to specific wards)

□ Providing various incentives for night, weekend, and holiday shifts, including additional compensation

□ Establishing an independent “Hospital medicine” and introducing a hospitalist training program

□ Ensuring faculty status

□ Other ( )
